# Use of phosphate-binders and risk of infection-related and all-cause mortality in patients undergoing hemodialysis: The Q-Cohort Study

**DOI:** 10.1038/s41598-018-29757-0

**Published:** 2018-07-30

**Authors:** Shunsuke Yamada, Masanori Tokumoto, Masatomo Taniguchi, Hisako Yoshida, Hokuto Arase, Narihito Tatsumoto, Hideki Hirakata, Takanari Kitazono, Kazuhiko Tsuruya

**Affiliations:** 1 Department of Medicine and Clinical Science, Graduate School of Medical Sciences, Kyushu University, Fukuoka, Japan; 2 Department of Internal Medicine, Fukuoka Dental College, Fukuoka, Japan; 3 Fukuoka Renal Clinic, Fukuoka, Japan; 4 Clinical Research Center, Saga University Hospital, Saga, Japan; 5 Department of Integrated Therapy for Chronic Kidney Disease, Graduate School of Medical Sciences, Kyushu University, Fukuoka, Japan

## Abstract

The use of phosphate (P)-binders allows hemodialysis patients to take in more protein and thus may maintain a good nutritional status. Protein-energy-malnutrition increases the risk of infection-related death. The association between use of P-binders and the relative risks of infection-related death remains unknown in hemodialysis patients. A total of 2926 hemodialysis patients registered to the Q-Cohort Study was followed up for 4-years. The association between use of P-binders and the risks for infection-related and all-cause mortality were estimated by Cox proportional hazards risk model with multiple adjustments by conventional and propensity-score based approaches. During the follow-up period, 106 patients and 492 patients died of infection and any cause, respectively. Cox proportional hazards models with multivariable adjustments including nutritional confounders showed that the incidence of infection-related death was significantly lower in patients with P-binders use compared with those without (hazard ratio [95% confidence interval] for infection-related mortality 0.63 [0.40–0.99]). The results remained significant even after applying four different propensity score-based analyses. Notably, use of P-binders was associated with a lower risk of all-cause mortality. Further studies including randomized controlled clinical trials and observational studies analyzed by an instrumental variable model will provide more robust evidences for the associations observed in our study.

## Introduction

Phosphate (P) overload, often presenting as hyperphosphatemia, is a typical manifestation of mineral and bone disorders and is highly prevalent in patients with chronic kidney disease (CKD)^1,2^. Accumulating evidence has shown that hyperphosphatemia is a direct cause of cardiovascular disorders in CKD patients^3–5^. Dietary P restriction and use of P-binders are currently the major therapeutic options for hyperphosphatemia^6,7^, both of which treatment modalities can improve P management in CKD patients. However, given that protein is a major source of dietary P intake, severe dietary protein restriction is often followed by dietary energy restriction and protein-energy-malnutrition. Protein-energy-malnutrition is known to increase the risks of morbidity and mortality in dialysis patients^6,8^. The use of P-binders thus appears to be a more practical approach for maintaining good nutritional status in the CKD population, with control of serum P levels within the recommended range.

Infection is the second-leading cause of death in hemodialysis patients^9,10^. Hemodialysis patients accumulate various risk factors that impair the immune system, putting them at increased risk of infection and its related mortality^11,12^. Among the numerous risk factors, malnutrition is a major contributor to the development and fatality of infections in CKD patients^13–15^. Because P-binders can improve P management with less risk of malnutrition than dietary protein restriction, these agents are expected to allow patients to maintain a better nutritional state whilst decreasing the chance of infection, thereby reducing the risk of infection-related mortality. However, the relative risks of infection-related death among hemodialysis patients with and without P-binders are largely unknown.

The present study aimed to compare the risks of infection-related mortality among patients undergoing hemodialysis with or without P-binders. We analyzed the dataset of the Q-Cohort Study, which was a prospective multicenter cohort study of hemodialysis patients in Japan, focusing on nutritional indicators as a potential cofounding factor^16–18^.

## Results

### Baseline characteristics of the participants

A total of 2409 patients (82.3%) received P-binders at baseline. The baseline characteristics of the 2926 patients with and without P-binders in the unmatched cohort are listed in Table 1. Patients with P-binders had a lower mean age, lower rate of diabetes mellitus (DM), lower incidence of comorbidities, longer median dialysis history, longer mean dialysis time per session, greater mean normalized protein catabolic rate (nPCR) and body mass index (BMI) values, lower cardiothoracic ratio, and higher systolic blood pressure. Mean blood hemoglobin and mean serum albumin, urea nitrogen, creatinine (Cr), corrected calcium, and P levels were higher in subjects with P-binders, whereas mean serum C-reactive protein and alkaline phosphatase levels and the frequency of erythropoiesis-stimulating agent administration were lower in patients with P-binders. P-binder users received vitamin D receptor activators more frequently. The average daily dose of P-binders in patients treated with P-binders was 2499 mg/day.

**Table 1:** Baseline clinical backgrounds of the patients treated with or without phosphate-binders.

Characteristics	Entire cohort (n = 2926)			
	Use of phosphate-binders		*P*-value	Standardized difference
	No (n = 517)	Yes (n = 2409)		
**Demographics and Comorbidities**				
Age, years	68.7 ± 12.6	62.8 ± 12.6	<0.001	0.469
Sex, male, %	56	60	0.085	0.084
Diabetes mellitus, %	35	28	0.002	0.148
Comorbidity, %	51	38	<0.001	0.254
Dialysis history, years	3.4 (0.9–9.1)	5.7 (2.4–11.6)	<0.001	0.214
Dialysis time per session, hours	4.6 ± 0.6	4.7 ± 0.6	<0.001	0.244
Kt/V for urea	1.58 ± 0.31	1.58 ± 0.30	0.903	0.006
nPCR, g/kg/day	0.90 ± 0.23	0.96 ± 0.19	<0.001	0.307
Body mass index, kg/m^2^	20.2 ± 3.5	21.3 ± 3.3	<0.001	0.314
Cardiothoracic ratio, %	51.3 ± 6.1	50.6 ± 5.4	<0.001	0.123
Systolic blood pressure, mmHg	151 ± 25	154 ± 23	<0.001	0.108
**Laboratory tests**				
Blood hemoglobin, g/dL	10.4 ± 1.2	10.6 ± 1.1	<0.001	0.159
Blood urea nitrogen, mg/dL	57.3 ± 16.0	67.8 ± 14.3	<0.001	0.690
Serum creatinine, mg/dL	8.4 ± 2.4	10.6 ± 2.6	<0.001	0.873
Serum albumin, g/dL	3.6 ± 0.5	3.8 ± 0.4	<0.001	0.577
Serum total cholesterol, mg/dL	151 (127–182)	151 (131–176)	0.874	0.009
Serum C-reactive protein, mg/dL	0.18 (0.07–0.50)	0.13 (0.05–0.26)	<0.001	0.258
Corrected serum Ca, mg/dL	9.3 ± 0.8	9.4 ± 0.8	<0.001	0.187
Serum phosphate, mg/dL	4.6 ± 1.2	5.0 ± 1.2	<0.001	0.343
Serum alkaline phosphatase, U/L	304 ± 161	258 ± 136	<0.001	0.313
Serum PTH (intact assay), pg/mL	100 (51–195)	102 (45–211)	0.295	0.054
**Medication**				
Use of ESAs, %	89	83	0.003	0.148
Use of anti-hypertensives, %	62	65	0.189	0.064
Use of VDRAs, %	61	73	<0.001	0.249
**Use of phosphate-binders**				
Sevelamer hydrochloride, %	0	35	<0.001	1.031
Ca-containing binders, %	0	86	<0.001	3.524
Total daily dose of phosphate-binders, g/day	0	2499 ± 1512	NA	NA

Data are expressed as the mean ± standard deviation, median (interquartile range), or percentage, depending on the nature of the variable. Unpaired *t*-test, chi-square test, or Wilcoxon sign-rank test was used to compare the two groups. Standardized difference was also calculated. A two-tailed *P*-value less than 0.05 was considered statistically significant. Comorbidity included history of cardiovascular events, bone fracture, and parathyroidectomy. Total daily dose of phosphate-binders was also calculated by adding daily dose of calcium carbonate and sevelamer hydrochloride and expressed as that of calcium carbonate. In the present study, Phosphate binding capacity of 750 mg of sevelamer hydrochloride was regarded equivalent to that of 500 mg of calcium carbonate. Abbreviations: Ca, calcium; ESAs, erythropoiesis-stimulating agents; NA, not applicable; nPCR, normalized protein catabolic rate; PTH, parathyroid hormone; VDRAs, vitamin D receptor activators.

**Figure 1:**
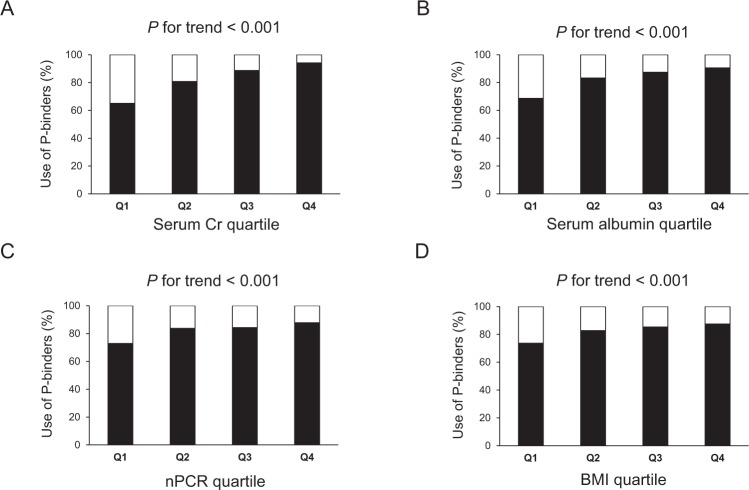
Percentage of phosphate-binders use in each quartile group stratified by nutritional indicators. (**A**) Serum Cr. (**B**) Serum albumin. (**C**) nPCR. (**D**) BMI. Patients were divided into quartiles based on serum Cr, serum albumin, nPCR, or BMI. Q1 is the lowest quartile and Q4 is the highest quartile. Cochran-Armitage trend analyses were used to determine the trend between use of phosphate-binders and each nutritional indicator quartile. A two-tailed *P*-value < 0.05 was considered to be statistically significant. Abbreviations: BMI, body mass index; Cr, creatinine; nPCR, normalized protein catabolic rate; Q, quartile.

We determined the association between use of P-binders and nutritional status by examining the percentage of P-binders use stratified by quartiles based on serum Cr, albumin, nPCR, or BMI (Fig. 1A–D). In this study, higher levels of these four parameters were regarded as indicators of good nutritional status. Patients with higher quartiles of serum Cr and albumin, nPCR, or BMI used P-binders more frequently (*P* for trend <0.001 for each nutritional indicator, Cochran–Armitage trend test).

### Effects of phosphate-binders on the risk of infection-related death

**Figure 2:**
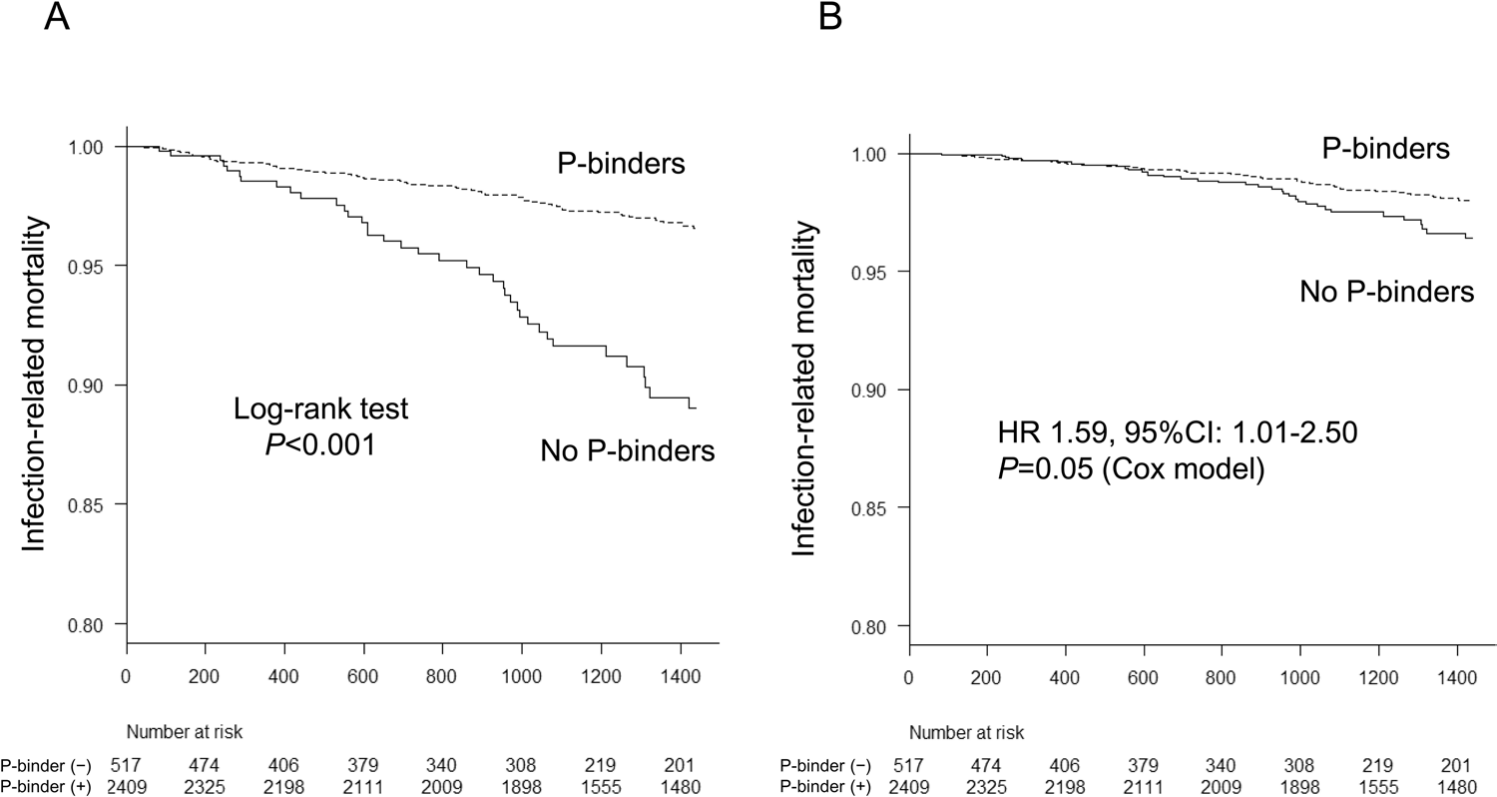
Kaplan-Meier analysis for the infection-related mortality between phosphate-binder users and non-users. (**A**) Non-adjusted curve. (**B**) Multivariable-adjusted curve. Log-rank test was used to compare the survival curve between the two groups in non-adjusted curve. Adjustment using Cox proportional hazard model was performed for baseline characteristics (age, sex, presence of diabetes mellitus and comorbidity, dialysis vintage, dialysis time per session, Kt/V for urea, normalized protein catabolic rate, body mass index, cardiothoracic ratio, systolic blood pressure, blood hemoglobin level, serum levels of urea nitrogen, creatinine, albumin, total cholesterol, C-reactive protein, corrected calcium, P, parathyroid hormone, alkaline phosphatase, and use of erythropoiesis stimulating agents, anti-hypertensives, and vitamin D receptor activators. The relative hazards risk and 95%Ci in patients without P-binders compared with those with P-binders were described in the figure. A two-tailed *P*-value < 0.05 was considered to be statistically significant. Abbreviations: CI, confidence interval; HR, hazard ratio; P, phosphate.

During a median follow-up period of 4 years, 106 patients died of infection, including 68 patients using P-binders and 38 non-users. The cumulative incidence rate of infection-related death expressed as 1000 person-year was 27.4 for non-P-binders users and 8.7 for P-binders users. Unadjusted and multivariable adjusted Kaplan–Meier curves showed a significantly higher rate of infection-related mortality in patients without P-binders compared with those with P-binders (log-rank test, *P* < 0.001) (Fig. 2). Unadjusted Cox proportional hazards analysis showed a significant association between the use of P-binders and a decreased risk of infection-related mortality (hazard ratio (HR) [95% confidence interval (CI)], 0.31 [0.21–0.46]; *P* < 0.001). Use of P-binders was independently associated with a lower incidence of infection-related death, even after adjusting for the potential confounding factors (Models 1 and 2). The association remained significant but weaker after adjusting for nutritional indicators (Model 3: HR [95% CI], 0.63 [0.40–0.99]; *P* = 0.049) (Table 2). The results also remained significant when risk estimates were assessed using a Fine–Gray subdistribution hazards model. Moreover, the total daily dose of P-binders was significantly associated with a decreased risk of infection-related death: multivariable adjusted HR [95% CI] for every 1 g/day increase in total daily dose of P-binders by Cox model (0.81 [0.68–0.97]; *P* = 0.023).

**Table 2:** Association between treatment with phosphate-binders and infection-related mortality (n = 2926).

Models	Unadjusted model		Model 1		Model 2		Model 3	
	HR (95% CI)	*P*-value	HR (95% CI)	*P*-value	HR (95% CI)	*P*-value	HR (95% CI)	*P*-value
Use of phosphate-binders								
Cox proportional hazard model	0.31 (0.21–0.46)	<0.001	0.46 (0.30–0.70)	0.004	0.47 (0.31–0.72)	0.007	0.63 (0.40–0.99)	0.049
Fine-Gray subdistribution hazards model	0.35 (0.23–0.52)	<0.001	0.53 (0.34–0.80)	0.003	0.53 (0.34–0.82)	0.004	0.64 (0.39–1.05)	0.078
Every 1 g/day increase in daily phosphate-binders dose								
Cox proportional hazard model	0.61 (0.52–0.72)	<0.001	0.72 (0.61–0.85)	<0.001	0.72 (0.61–0.85)	<0.001	0.81 (0.68–0.97)	0.023
Fine-Gray subdistribution hazards model	0.64 (0.54–0.76)	<0.001	0.75 (0.63–0.89)	0.001	0.75 (0.63–0.89)	0.001	0.82 (0.69–0.98)	0.031

The risk estimates are expressed as HR (95%CI). The HR was estimated using Cox proportional hazard model for conventional approach and Fine-Gray subdistribution hazards model for dealing with competing risk. The following covariates were included in each model: Model 1, age, sex, presence of diabetes mellitus and comorbidity, dialysis vintage, dialysis time per session, Kt/V for urea; Model 2, covariates in Model 1, cardiothoracic ratio, systolic blood pressure, blood hemoglobin level, serum levels of corrected calcium, phosphate, alkaline phosphatase, parathyroid hormone, use of erythropoiesis stimulating agents, anti-hypertensives, and vitamin D receptor activators; Model 3, covariates in Model 2, normalized protein catabolic rate, body mass index, serum levels of urea nitrogen, creatinine, albumin, total cholesterol, and C-reactive protein. Total daily dose of phosphate-binders was expressed as that of calcium carbonate. In this study, Phosphate binding capacity of 750 mg of sevelamer dose was regarded equivalent to that of 500 mg of calcium carbonate. A two-tailed *P*-value less than 0.05 was considered statistically significant. Abbreviations: CI, confidence interval; HR, hazard ratio.

### Effects of phosphate-binders on the risk of infection-related death under propensity score-based adjustments

To rule out confounding by indication of prescription at baseline, we performed propensity score (PS)-adjusted analyses using four different approaches. We used PS to create a matching cohort for the use of P-binders. The propensity model showed good discriminatory ability and was well-calibrated (C-statistics = 0.78, Hosmer test; *χ*^2^ statistic 6.35; *P* = 0.61). Imbalances in baseline covariates including nutritional indicators in the pre-matching cohort were well-balanced after PS matching, with standardized differences in each covariate <0.1 (Supplementary Table 1). Notably, the PS-matched cohort showed a significantly lower incidence of infection-related death in patients treated with P-binders (HR [95%CI], 0.58 [0.34–0.98]; *P* = 0.042) (Table 3). Furthermore, patients treated with P-binders had greater infection-related survival than those without P-binders, even after PS-based analysis including PS stratification, PS adjustment, and inverse probability of treatment weighting (IPTW).

**Table 3:** Association between treatment with phosphate-binders and infection-related mortality.

PS-adjusted model	Infection-related mortality	
	HR (95% CI)	*P*-value
PS-matched model (1:1, n = 928)	0.58 (0.34–0.98)	0.042
PS-stratification (n = 2926)	0.56 (0.37–0.85)	0.007
PS-adjusted regression model (n = 2926)	0.59 (0.34–0.93)	0.022
IPTW model (n = 2926)	0.63 (0.40–0.98)	0.040

The HR was estimated using Cox proportional hazard model. In the multivariable model, age, sex, presence of diabetes mellitus and comorbidity, dialysis history, dialysis time per session, Kt/V for urea, normalized protein catabolic rate, body mass index, cardiothoracic ratio, systolic blood pressure, blood hemoglobin level, serum levels of urea nitrogen, creatinine, albumin, total cholesterol, C-reactive protein, corrected calcium, phosphate, alkaline phosphatase, and parathyroid hormone, and use of erythrocyte stimulating agents, anti-hypertensives, and vitamin D receptor activators were included. PS was created by logistic regression analysis using all the parameters listed here. A two-tailed *P*-value less than 0.05 was considered statistically significant. Abbreviations: CI, confidence interval; HR, hazard ratio; IPTW, inverse probability of treatment weighting; PS, propensity score.

### Effect modifications by baseline characteristics regarding the association between use of phosphate-binders and incidence of infection-related death

We determined the effect modifications in subgroups stratified by baseline characteristics regarding the association between use of P-binders and the incidence of infection-related death by IPTW-based multivariable analysis. There were no significant interactions between baseline characteristics and use of P-binders (*P* = 0.074–0.691) (Supplementary Fig. 1).

### Effects of phosphate-binders on risk of all-cause death

**Figure 3:**
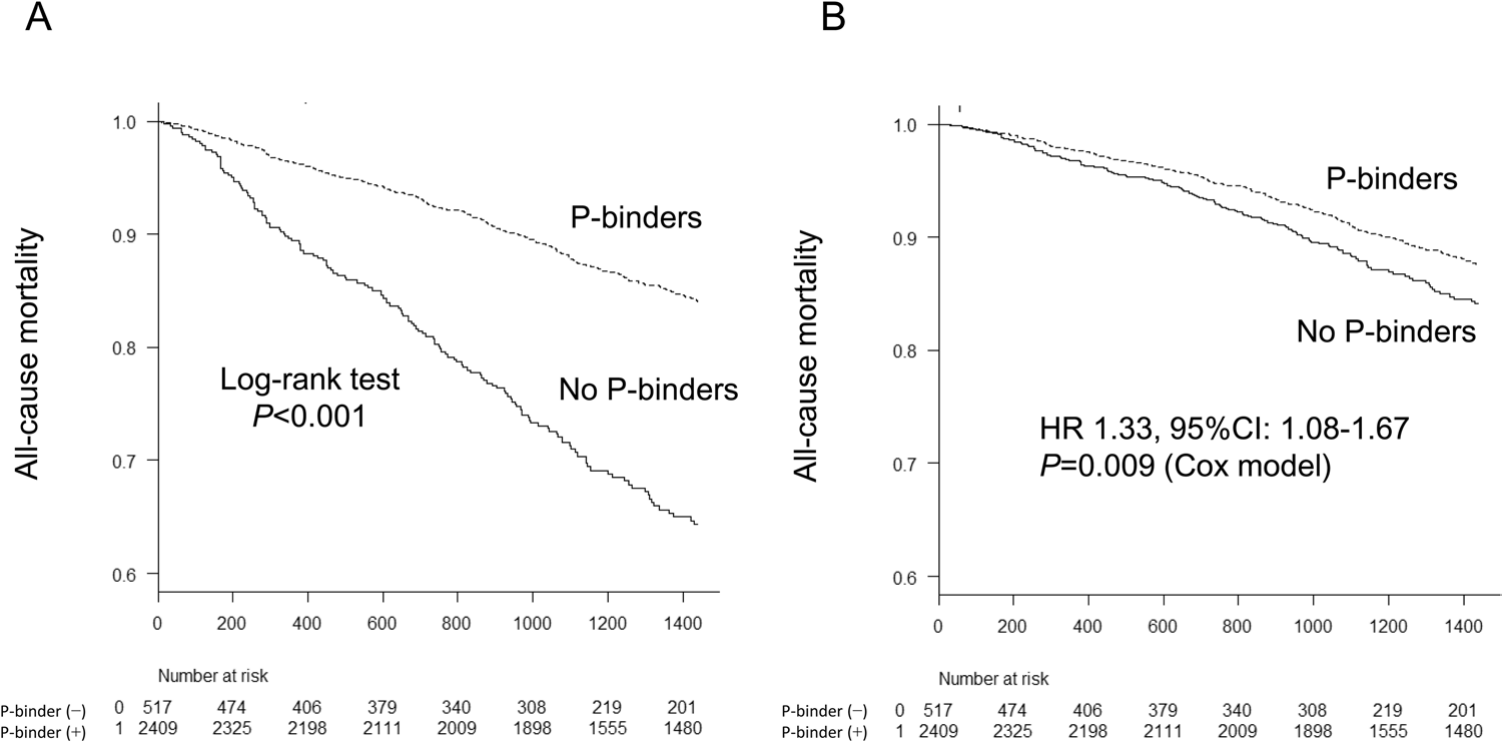
Kaplan-Meier analysis for the all-cause mortality between phosphate-binder users and non-users. (**A**) Non-adjusted curve. (**B**) Multivariable-adjusted curve. Log-rank test was used to compare the survival curve between the two groups in non-adjusted curve. Adjustment using Cox proportional hazard model was performed for baseline characteristics (age, sex, presence of diabetes mellitus and comorbidity, dialysis vintage, dialysis time per session, Kt/V for urea, normalized protein catabolic rate, body mass index, cardiothoracic ratio, systolic blood pressure, blood hemoglobin level, serum levels of urea nitrogen, creatinine, albumin, total cholesterol, C-reactive protein, corrected calcium, phosphate, parathyroid hormone, alkaline phosphatase, and use of erythropoiesis stimulating agents, anti-hypertensives, and vitamin D receptor activators. The relative hazards risk and 95%Ci in patients without P-binders compared with those with P-binders were described in the figure. A two-tailed *P*-value < 0.05 was considered to be statistically significant. Abbreviations: CI, confidence interval; HR, hazard ratio; P, phosphate.

We also determined the impact of P-binders on all-cause mortality. During a median follow-up period of 4 years, 492 patients died of any cause, including 338 P-binder users and 154 P-binder non-users. Unadjusted and multivariable adjusted Kaplan–Meier analyses showed that patients with P-binders had a significantly (*P* < 0.05) longer survival than patients without P-binders (Fig. 3). Unadjusted and multivariable-adjusted Cox proportional hazards analyses revealed that use of P-binders conferred a significant survival benefit over non-use (multivariable adjusted HR [95%CI], 0.75 [0.60–0.93]; *P* = 0.009) (Table 4). The results remained significant using all four different PS-based approaches. Moreover, the total daily dose of P-binders was significantly associated with a decreased risk of infection-related death: multivariable adjusted HR [95%CI] for every 1 g/day increase in total daily dose of P-binders by Cox model (0.91 [0.84–0.98], *P* = 0.009).

**Table 4:** Association between treatment with phosphate-binders and all-cause mortality.

PS-adjusted model	All-cause mortality	
	HR (95% CI)	*P*-value
Unadjusted model (n = 2926)	0.38 (0.32–0.46)	<0.001
Multivariable-adjusted model (n = 2926)	0.75 (0.60–0.93)	0.009
PS-adjusted model		
PS-matched model (1:1, n = 928)	0.74 (0.57–0.96)	0.022
PS-stratification (n = 2926)	0.74 (0.58–0.96)	0.021
PS-adjusted regression model (n = 2926)	0.74 (0.59–0.92)	0.006
IPTW model (n = 2926)	0.69 (0.53–0.91)	0.007

The HR was estimated using Cox proportional hazard model. In the multivariable model, age, sex, presence of diabetes mellitus and comorbidity, dialysis history, dialysis time per session, Kt/V for urea, normalized protein catabolic rate, body mass index, cardiothoracic ratio, systolic blood pressure, blood hemoglobin level, serum levels of urea nitrogen, creatinine, albumin, total cholesterol, C-reactive protein, corrected calcium, phosphate, alkaline phosphatase, and parathyroid hormone, and use of erythrocyte stimulating agents, anti-hypertensives, and vitamin D receptor activators were included. PS was created by logistic regression analysis using all the parameters listed here. A two-tailed *P*-value less than 0.05 was considered statistically significant. Abbreviations: CI, confidence interval; HR, hazard ratio; IPTW, inverse probability of treatment weighting; PS, propensity score.

The effect modifications in subgroups stratified by baseline characteristics were also determined regarding the association between the use of P-binders and all-cause mortality using IPTW-based multivariable analysis. There were no significant interactions between baseline characteristics and use of P-binders (*P* = 0.051–0.741) (Supplementary Fig. 2).

### Sensitivity analyses

To further determine the dose response relationship between use of P-binders and outcomes, we divided our patients into three groups based on the dose of P-binders at baseline and compared the risks for patient outcomes among the three groups. As shown in Supplementary Table S2, survival in relation to infection-related and all-cause mortality was better in patients taking P-binders Group 2 (0 mg/dL < P-binders < 2000 mg/day) and Group 3 (2000 mg/day ≤ P-binders ≤ 15000 mg/day) compared with patients without P-binders Group 1 (P-binders; 0 mg/day), and a higher dose of P-binders (Group 3) was associated with a greater reduction in mortality.

To elucidate whether there is a beneficial impact of sevelamer hydrochloride over calcium-based P-binders on outcomes, we created PS-matched cohort (1:1, n = 566) and compared the risk for patient outcomes between patients treated only with calcium-based P-binders and those treated only with sevelamer hydrochloride. However, there was no significant risk reduction in patients treated only with sevelamer hydrochloride compared with those treated only with calcium-based P-binders: risk for infection-related death, 1.04 [0.40–2.65], *P* = 0.94; risk for all-cause death, 1.04 [0.40–2.65], *P* = 0.63 (versus calcium-based P-binders users).

We also examined the beneficial impact of P-binders on mortality after excluding patients with baseline serum P levels ≤3 mg/dL, since hypophosphatemic patients are less likely to receive P-binders. As shown in Supplemental Table 3, even after excluding patients with lower serum P levels, PS-based analysis demonstrated significant or marginally significant associations between treatment with P-binders and a reduced risk of infection-related and all-cause mortality (Supplementary Table 3).

## Discussion

The present observational study indicated that hemodialysis patients treated with P-binders had lower risks of infection-related and all-cause deaths compared with patients without P-binders, independent of their baseline clinical characteristics and even after rigorous adjustment for nutritional indicators. The results remained significant even after applying PS-based approaches to rule out potential confounding by treatment indication at baseline, although the associations were significantly attenuated after adjustments for nutritional factors. These results suggest that hemodialysis patients treated with P-binders benefit from lower risks of infection-related and all-cause deaths, possibly by maintaining a good nutritional status.

Accumulating evidence has shown that P-binders have favorable impacts on mortality^19,20^. P overload is closely related to the pathogenesis of diverse cardiovascular disorders, and the underlying biological mechanisms responsible for the benefit of P-binders have thus been explained in terms of decreased cardiovascular morbidity and mortality^3,5,21,22^. Notably however, both infection-related and all-cause mortalities were lower in patients treated with P-binders in the present study. To the best of our knowledge, this observation provides the first evidence indicating that P-binders could decrease the risk of death in hemodialysis patients by lowering the incidence of infection-related deaths.

Nutritional status is a major contributor to the risks of infection and infection-related hospitalization and mortality in hemodialysis patients^13,15^. It is likely that P-binders tend to be prescribed to patients with good nutritional status, because they are more likely to be healthy individuals with a good appetite and thus have higher serum P levels. Indeed, P-binders were more frequently prescribed to patients with higher levels of nutritional indicators at baseline in the present study, indicating that the observed association between the use of P-binders and the risk of infection-related mortality was confounded by nutritional status. Actually, the HR of P-binders use was attenuated by adjustment for nutritional factors (Model 3) (Table 2). These results show that it was difficult to separate the tight link between P-binders use and good nutritional status in the observational study. However, the associations remained statistically significant even after rigorous adjustments for covariates related to nutritional status in multivariable analysis including PS-based approaches. Furthermore, we recently demonstrated that P-binders prevented malnutrition and inflammation, while dietary protein restriction induced malnutrition in an animal study^23,24^. Taken together, these results suggest that the use of P-binders may decrease the risk of infection-related death independent of the patient’s baseline nutritional status, probably by maintaining a better nutritional status during the observation period. Actually, a very recent study showed that patients newly treated with lanthanum carbonate have a nutritional benefit by staying at a good nutritional status and survived longer than those not treated by lanthanum carbonate in maintenance hemodialysis patients^25^. This paper strongly supports our hypothesis that maintenance of good nutritional status will contribute to the decreased risk of infection-related and all-cause death in patients treated with P-binders. However, the nature of this observational study means that it is difficult to determine if patients treated with P-binders are able to maintain good nutritional status, or if patients in a good nutritional condition required P-binders to control serum P levels. Also, there may be other mechanisms other than nutritional benefit that explain the decreased risk of infection-related death in patients treated with P-binders. Further studies are therefore needed to address the clinically important issue of whether P-binders can provide nutritional and non-nutritional benefits and decrease the risk of infection-related death in hemodialysis patients.

The terms ‘malnutrition-inflammation-cachexia/complex syndrome’ and ‘malnutrition-inflammation-atherosclerosis syndrome’ are widely recognized in the CKD population^26,27^. These concepts are corroborated by the interactive nature of nutritional status and inflammation, leading to a vicious cycle and affecting all-cause mortality^28^. Importantly, increasing evidence has shown the involvement of P overload in the pathogenesis of systemic inflammation in CKD. P loading dose-dependently induced systemic inflammation and malnutrition in uremic rats and vascular smooth muscle cells^23,29^. Calciprotein particles, which increase in response to relative P overload, were shown to act on lymphocytes, monocytes, and vascular smooth muscle cells and induce an inflammatory response^30,31^. Furthermore, clinical studies showed that fibroblast growth factor 23 (FGF23), which increases in response to P loading, was closely associated with serum inflammatory markers and an increased risk of infection-related hospitalization in hemodialysis patients^32,33^. Basic studies revealed that FGF23 directly inhibited neutrophil recruitment and calcitriol synthesis in macrophages, both of which are critical for the prevention of infectious diseases in hemodialysis patients^34,35^. Moreover, FGF23 acted on hepatocytes to produce inflammatory cytokines^36^. Considering that P-binders attenuate P overload and decrease FGF23 levels^37^, their use is recommended in patients with hyperphosphatemia, given that they can prevent P overload-induced systemic inflammation, block the vicious cycle of inflammation and malnutrition, inhibit impairments in immune function, and thereby reduce the risk of infection-related mortality in patients undergoing hemodialysis. However, because these suggested mechanisms are based on our speculation and results of the subgroup analysis indicated a possible confounding by baseline nutritional status even after multiple adjustments, our results should be interpreted with great caution and confirmed by further studies including basic research.

Increasing clinical studies have shown the favorable impact of P-binder use on survival^38–41^. A recent observational study showed that dialysis patients administered P-binders had 29% lower all-cause mortality compared with those without P-binders, which was compatible with our observations^41^. However, the beneficial effects of P-binders on all-cause mortality remain controversial. Indeed, a recent meta-analysis of randomized controlled trials (RCTs) concluded that there was no evidence that P-binders reduced the risk of all-cause mortality compared with placebo^42^. However, it is possible that this meta-analysis failed to detect an advantage of P-binders because of the relatively short study periods. Given that the benefits of P-binders are exerted through cardio- and infection-protective effects, most previous RCTs were too short to detect any impact on all-cause mortality. In this regard, observational studies with relatively longer study periods, such as the current study, can provide clinically significant results as long as critical biases and confounding factors are handled appropriately^43^.

Another interesting subject would be whether the impacts of P-binders use on infection-related mortality was different across the type of vascular access. Given that patients treated with dialysis catheters and arteriovenous grafts are more prone to develop vascular access-related infection than those treated with arteriovenous fistula^44^, it is probable that P-binders may reduce the incidence of infection of vascular access and infection-related death. Unfortunately, we did not have any information on the type of vascular access in the current study. Future large-scale studies focusing on the associations between treatment with P-binders and the reduced risk for the incidence of infection and infection-related death are needed to confirm our hypothesis.

This study had several strengths, including a relatively large number of patients and prospectively collected baseline data. The baseline characteristics included various serum biochemical parameters, and possible confounding factors were vigorously adjusted by conventional and PS-based statistical techniques to manage confounding by indication^17^. However, the study also had several limitations. First, the nature of the study meant that we were unable to confirm causality between P-binders use and infection-related death. Second, we assessed the use of P-binders only at baseline and did not have access to any longitudinal data during the observation period. So cross-over prescription might have biased the results regarding the impact of P-binders use on mortality. This is one of the critical limitations of our study. Hence, our hypothesis should be confirmed by time-dependent analyses in the future. Third, PS should be created by the data obtained before treatment with P-binders was initiated. Unfortunately, we did not have access to those data and created PS based on the baseline data, indicating that our PS-based analysis was not sufficient to handle the potential measured confounders and our observation might have been biased. Forth, although the impact of dialysis facility on the association between P-binders use and mortality was accounted in the current study, we cannot exclude the possibility that the difference in the practice pattern across participating dialysis facilities might have biased the association between P-binders use and mortality and our observation may shift towards null hypothesis once these potential biases have been accounted for. Fifth, we had no data on the incidence of infection, and only analyzed infection-related mortality. It therefore remains unclear if P-binders can reduce the incidence of infection, or just the risk of fatalities due to infection. In addition, it may be difficult to conclude events correctly and some patients might have been misclassified as suffering infection-related death or other cause of death. In this regard, our case might be confounded by misclassification bias. Finally, although we adjusted vigorously for both known and unknown potential confounding factors using PS-based approaches, we were unable to rule out completely the possibility that unmeasured and residual confounding factors, including serum FGF23 levels, might have biased the observed association between the use of P-binders and the reduced risks of mortality^45^, because subgroup analyses (Supplementary Fig. S1) indicated the presence of effect modifications in some of the baseline nutritional indicators such as BMI, DM, and serum albumin level and because we just adjusted the baseline nutritional parameters and did not take into account the nutritional status during the observation period. Importantly, patients with a good nutritional status tend to stay in the good nutritional condition and vice versa. These results suggest that the decreased risk of infection-related death by P-binders use might be still confounded by nutritional factors. With all these limitation, we believe that our current observation will provide medical practitioners with valuable information in the management of hyperphosphatemia and increased mortality in hemodialysis patients. However, the results of this study should be interpreted with caution and confirmed by future studies that can handle our potential limitations such as randomized controlled clinical trials and observational studies with an instrumental variable model.

In conclusion, our results suggest that use of P-binders leads to lower risks of infection-related and all-cause deaths in patients undergoing hemodialysis, even after PS-based multivariable adjustment for potential confounding factors, including nutritional indicators. Further studies, including RCTs with long observation periods, are needed to determine if P-binders use can decrease the incidence of infection and its related mortality. Until then, our observation should be interpreted with caution.

## Methods

### Design of the Q-Cohort Study and study subjects

The Q-Cohort Study was a multicenter, prospective, longitudinal observational study designed to identify risk factors for morbidity and mortality in patients undergoing hemodialysis^16–18,46^. Briefly, the study population consisted of 3598 outpatients aged ≥18 years, who underwent regular hemodialysis therapy between December 2006 and December 2007 at 39 dialysis facilities in Fukuoka and Saga Prefectures in Kyushu Island, located in the western part of Japan. All the patients were followed up until December 2010, unless they were lost to follow-up.

Measured outcomes were cause of mortality (infection-related, tumor-related, and all-cause of mortality), major cardiovascular events, which were defined as first-ever development of cardiovascular death, stroke, myocardial infarction, hospitalization for unstable angina, coronary intervention (coronary artery bypass surgery or angioplasty), hospitalization for heart failure, and/or peripheral vascular diseases, parathyroidectomy, and bone fracture. All events were adjudicated on the basis of patient medical records and imaging performed by the study members.

Among the 3598 patients registered to the study, 127 patients were excluded from the current analysis because of missing outcome data, and 545 patients were excluded because of insufficient information on their baseline characteristics and medications. A total of 2926 patients were therefore analyzed in the present study. The study was performed according to the Ethics of Clinical Research (Declaration of Helsinki). The study protocol was approved by the Kyushu University Hospital Institutional Review Board for Clinical Research (No. 20–31), and was registered in the clinical trial registry (University Hospital Medical Information Network, UMIN000000556). All patients provided written informed consent prior to study participation.

### Definition of outcomes and phosphate-binder exposure

The primary outcome was infection-related death and the secondary outcome was all-cause death. We defined “infection-related death” as death due to infections including septicemia, respiratory (e.g. pneumonia), abdominal and gastrointestinal (e.g. appendicitis, diverticulitis), cardiac (e.g. endocarditis), kidney and genitourinary (e.g. pyelonephritis, pelvic inflammatory disease), neurologic (meningitis), access-related, and other infections. The main exposure was use of P-binders at baseline. Patients were divided into those treated with and without P-binders. The total daily dose of P-binders was calculated by adding the daily doses of calcium carbonate and sevelamer hydrochloride, and expressed as dose of calcium carbonate, with the P-binding capacity of 750 mg of sevelamer hydrochloride regarded as equivalent to that of 500 mg of calcium carbonate.

### Covariates and biochemical determination

Baseline characteristics and potential confounding factors at baseline were collected by reviewing medical records. Briefly, the demographic information (age, sex, and dialysis vintage), comorbidity and history of treatment and disease events (presence of DM and histories of parathyroidectomy, cardiovascular events, and bone fracture), baseline clinical characteristics (height, body weight, BMI, nPCR, Kt/V for urea, systolic and diastolic blood pressure levels), dialysis-related information (dialysis session time and dialysate calcium concentration), and serum/blood biochemical parameters (blood hemoglobin level, blood hematocrit, serum levels of urea nitrogen, Cr, albumin, C-reactive protein, ferritin, total cholesterol, calcium, P, alkaline phosphatase, and parathyroid hormone [PTH]), and medications (use of erythropoiesis stimulating agents, anti-hypertensives, vitamin D receptor activators, and P-binders). In the current study, comorbidities included histories of cardiovascular events, bone fractures, and parathyroidectomy. Routine biochemical parameters were measured with an auto-analyzer using standard procedures. The corrected serum calcium concentration was adjusted depending on the serum albumin level^47^. Serum PTH level was measured using whole or intact PTH assays and the values measured by the two assays were interchnaged using the following equation: intact PTH (pg/mL) = 1.7 x  whole PTH (pg.mL)^48^. Target ranges of serum calcium, P, and PTH levels during the follow-up period were as follows; calcium, 8.4–10.0 mg/dL, P 3.5–6.0 mg/dL, intact PTH 60–180 pg/m^48^.

### Statistical analysis

Normally-distributed continuous variables, non-normally distributed continuous variables, and categorical variables were described as mean (standard deviation), median (interquartile range), or percentage. We compared baseline characteristics and laboratory data between the two groups using *χ*^2^, unpaired *t*, and Wilcoxon’s rank-sum tests.

We determined the associations between nutritional indicators and use of P-binders at baseline using serum levels of Cr and albumin, nPCR, and BMI as nutritional indicators. The patients were then divided into quartiles (Q1–Q4) based on serum Cr, serum albumin, nPCR, or BMI, respectively.

We also examined the estimated risks for infection-related and all-cause deaths between patients treated with and without P-binders using Cox proportional hazards models. Adjustment for potential confounding factors was performed sequentially: non-adjusted model, Model 1 (age, sex, presence of DM and comorbidity, dialysis vintage, dialysis time per session, and Kt/V for urea); Model 2 (covariates in Model 1, cardiothoracic ratio, systolic blood pressure, blood hemoglobin level, serum levels of corrected calcium, P, alkaline phosphatase, PTH, and use of erythropoiesis-stimulating agents, anti-hypertensives, and vitamin D receptor activators); and Model 3 (covariates in Model 2, nPCR, BMI, serum levels of urea nitrogen, Cr, albumin, total cholesterol, and C-reactive protein). Adjusted hazard risks were expressed as HR and 95% CIs. After setting non-infection-related death as a competing risk, we conducted the same analysis using a Fine–Gray subdistribution hazards model. The adjusted hazard risk for every 1 g/day increase in total daily P-binders dose was also calculated with respect to infection-related and all-cause deaths.

We minimized potential confounding and selection biases in this observational study by comparing the infection-related and all-cause mortality rates between patients with and without P-binders at baseline using PS adjustment^17^. The propensity for each category of P-binder use was determined by multivariable-adjusted logistic regression analysis using the variables listed above. The association between P-binders treatment and infection-related or all-cause mortality was also estimated using a Cox proportional hazards model stratified by PS quintile. Multivariable analysis including PS as a covariate was performed. Finally, IPTW was also analyzed using PS.

To detect potential heterogeneity in the effects of P-binders across baseline characteristics, a multiplicative interaction term was added to the relevant IPTW-based Cox regression model. As sensitivity analyses, to further determine the dose-response relationships between P-binders use and patient outcomes, patients were divided into three groups based the dose of P-binders. To elucidate whether sevelamer hydrochloride has beneficial impacts on mortality compared with calcium-based P-binders, we compared the risk for patient outcomes between patients treated with calcium-based P-binders and those treated with sevelamer hydrochloride regarding both outcomes. We also determined the impact of P-binders use on mortality after excluding patients with low serum P level (≤3 mg/dL). A two-tailed *P*-value < 0.05 was considered statistically significant in all analyses. Statistical analyses were performed using the JMP version 13.2 software program (SAS Institute Inc., Tokyo, Japan) and R version 3.0.2 (http://cran.rproject.org).

### Data availability

The datasets generated during and/or analyzed during the current study are available from the corresponding author on reasonable request.

## Electronic supplementary material

Supplementary information
