# Supplementary material for: Use of phosphate-binders and risk of infection-related and all-cause mortality in patients undergoing hemodialysis: The Q-Cohort Study
Source: Sci Rep. 2018 Jul 30;8:11387. doi: 10.1038/s41598-018-29757-0 (PMC6065422; doi:10.1038/s41598-018-29757-0)
Supplement: Supplementary file 1 — Supplementary information [file 41598_2018_29757_MOESM1_ESM.docx]

**Supplementary Information**

**Use of phosphate-binders and risk of infection-related and all-cause mortality in patients undergoing hemodialysis: The Q-Cohort Study**

Shunsuke Yamada MD, PhD ^1^; Masanori Tokumoto MD, PhD ^2^; Masatomo Taniguchi MD, PhD ^3^; Hisako Yoshida, PhD ^4^; Hokuto Arase MD^1^; Narihito Tatsumoto MD, PhD ^1^; Hideki Hirakata MD, PhD ^3^; Takanari Kitazono, MD, PhD ^1^; *Kazuhiko Tsuruya MD, PhD ^1,5^

1. Department of Medicine and Clinical Science, Graduate School of Medical Sciences, Kyushu University, Fukuoka, Japan

2. Department of Internal Medicine, Fukuoka Dental College, Fukuoka, Japan

3. Fukuoka Renal Clinic, Fukuoka, Japan

4. Clinical Research Center, Saga University Hospital, Saga, Japan

5. Department of Integrated Therapy for Chronic Kidney Disease, Graduate School of Medical Sciences, Kyushu University, Fukuoka, Japan

Running title: Phosphate-binders and infection-related death

*Address for correspondence:*

*Kazuhiko Tsuruya, MD, PhD

Department of Integrated Therapy for Chronic Kidney Disease,

Graduate School of Medical Sciences, Kyushu University

3-1-1 Maidashi, Higashi-ku, Fukuoka 812-8582, Japan,

Tel: +81-92-642-5843 Fax: +81-92-642-5846,

E-mail: [tsuruya@intmed2.med.kyushu-u.ac.jp](mailto:tsuruya@intmed2.med.kyushu-u.ac.jp)

**Supplementary Figure Legends**

**
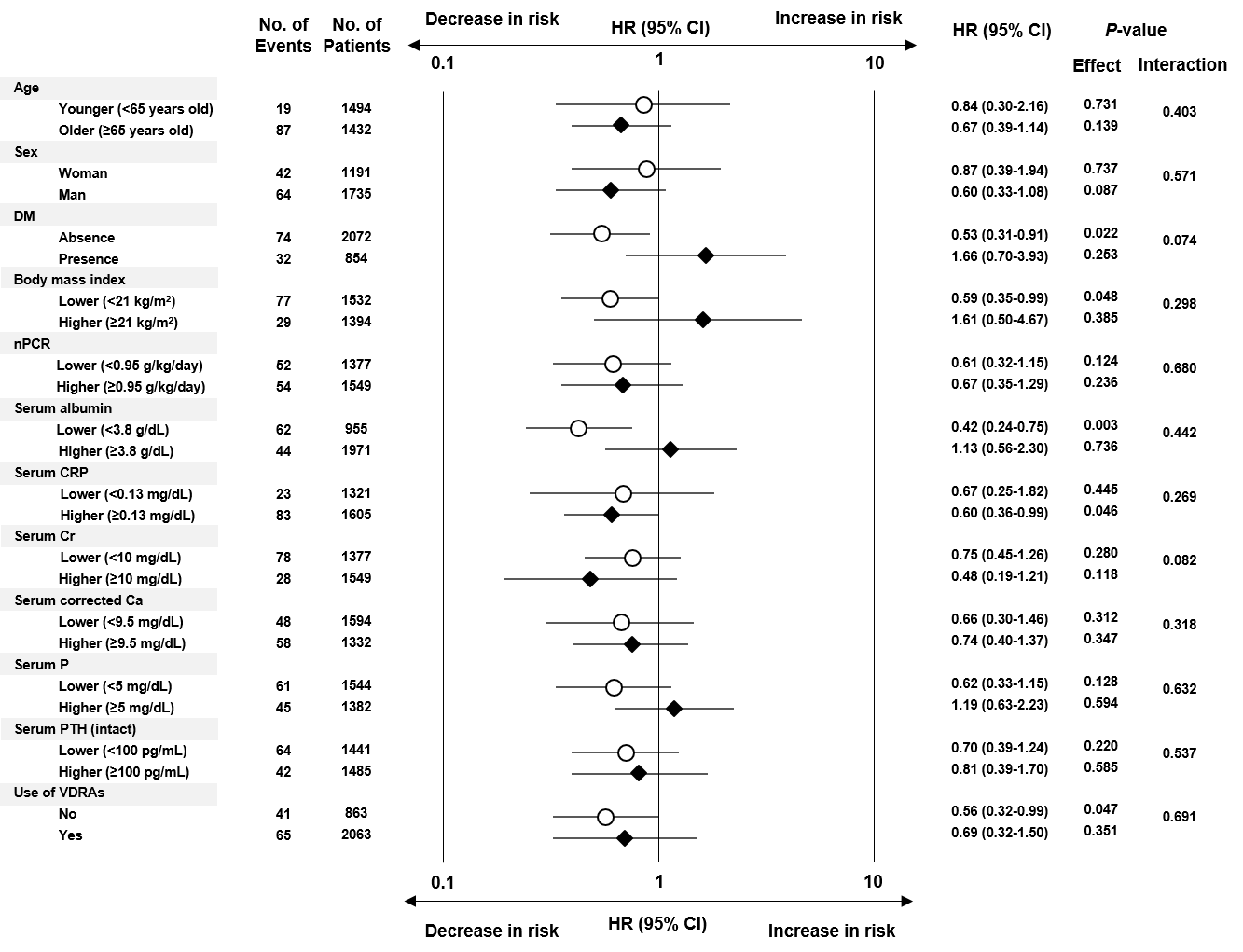
**

**Supplemental Figure 1**. PS-weighted HRs and 95%CI for the effect of P-binders on infection related death in each group stratified according the baseline characteristics and treatment. The estimated risk was adjusted for Cox proportional hazard risk model in PS-weighted samples. Adjustment was performed for baseline characteristics (age, sex, presence of DM and comorbidity, dialysis vintage, dialysis time per session, Kt/V for urea, nPCR, body mass index, cardiothoracic ratio, systolic blood pressure, blood hemoglobin level, serum levels of urea nitrogen, Cr, albumin, total cholesterol, CRP, corrected Ca, P, PTH, alkaline phosphatase, and use of erythropoiesis stimulating agents, anti-hypertensives, and VDRAs. Open circles and filled rhombus denote point estimate of HRs and error bars represent 95% CI. The results were adjusted using the final selected model. Variables relevant to the subgroups were excluded from each model. A two-tailed *P* value <0.05 was considered statistically significant. Abbreviations: Ca, calcium; CI, confidence interval; Cr, creatinine; CRP, C-reactive protein; DM, diabetes mellitus; HR, hazard ratio; nPCR, normalized protein catabolic rate; PS, propensity score; PTH, parathyroid hormone; VDRAs, vitamin D receptor activators.

**
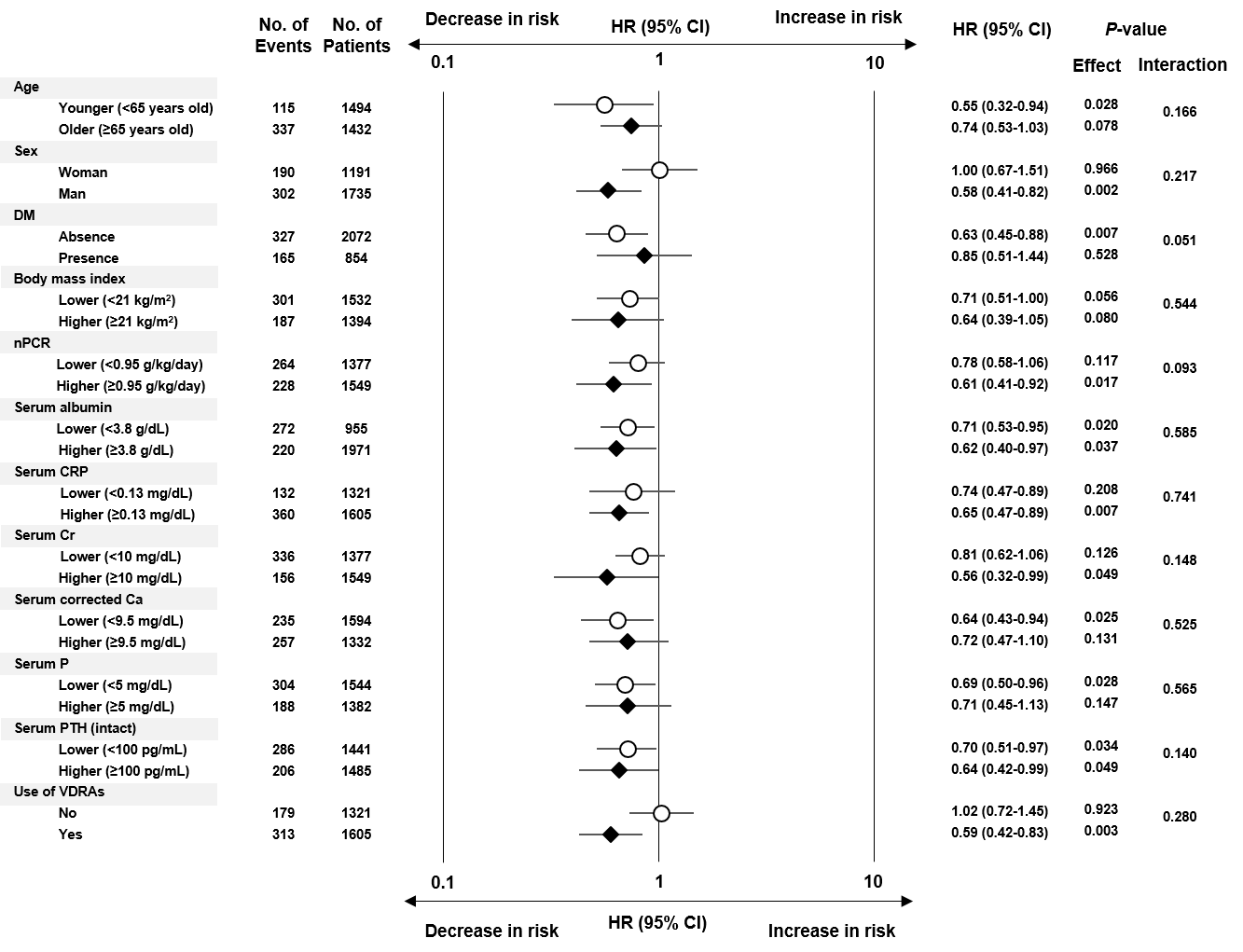
**

**Supplemental Figure 2**. PS-weighted HRs and 95%CI for the effect of P-binders on all-cause death in each group stratified by baseline characteristics and treatment. The estimated risk was adjusted for Cox proportional hazard risk model in PS-weighted samples. Adjustment was performed for baseline characteristics (age, sex, presence of DM and comorbidity, dialysis vintage, dialysis time per session, Kt/V for urea, nPCR, body mass index, cardiothoracic ratio, systolic blood pressure, blood hemoglobin level, serum levels of urea nitrogen, Cr, albumin, total cholesterol, CRP, corrected Ca, P, PTH, alkaline phosphatase, and use of erythropoiesis stimulating agents, anti-hypertensives, and VDRAs. Open circles and filled rhombus denote point estimate of HRs and error bars represent 95% CI. The results were adjusted using the final selected model. Variables relevant to the subgroups were excluded from each model. A two-tailed *P* value <0.05 was considered statistically significant. Abbreviations: Ca, calcium; CI, confidence interval; Cr, creatinine; CRP, C-reactive protein; DM, diabetes mellitus; HR, hazard ratio; nPCR, normalized protein catabolic rate; PS, propensity score; PTH, parathyroid hormone; VDRAs, vitamin D receptor activators.

# Supplementary Table 1. Baseline clinical backgrounds of the patients treated with or without phosphate-binders in propensity score-matched cohort

|  | Propensity score-matched cohort (n=928) | | | |
| --- | --- | --- | --- | --- |
| Characteristics | Use of phosphate-binders | | *P*-value | Standardized difference |
|  | No (n=464) | Yes (n=464) |  |  |
| **Demographics and Comorbidities** |  |  |  |  |
| Age, years | 68.2±12.6 | 68.6±11.9 | 0.674 | 0.038 |
| Sex, male | 57 | 58 | 0.894 | 0.017 |
| Diabetes mellitus, % | 35 | 33 | 0.530 | 0.046 |
| Comorbidity, % | 49 | 49 | 0.743 | 0.004 |
| Dialysis history, years | 3.8 (1-10.2) | 4.5 (1.6-9.6) | 0.999 | 0.015 |
| Dialysis time per session, hours | 4.6±0.6 | 4.6±0.6 | 0.675 | 0.038 |
| Kt/V for urea | 1.58±0.3 | 1.57±0.3 | 0.476 | 0.039 |
| nPCR, g/kg/day | 0.91±0.23 | 0.91±0.22 | 0.718 | 0.002 |
| Body mass index, kg/m^2^ | 20.5±3.5 | 20.5±3.3 | 0.610 | 0.016 |
| Cardiothoracic ratio, % | 51.2±6.0 | 51.5±5.8 | 0.285 | 0.044 |
| Systolic blood pressure, mmHg | 152±24 | 153±23 | 0.593 | 0.032 |
| **Laboratory tests** |  |  |  |  |
| Blood hemoglobin, g/dL | 10.4±1.2 | 10.4±1.2 | 0.736 | 0.019 |
| Blood urea nitrogen, mg/dL | 59.2±15.2 | 58.9±14.3 | 0.736 | 0.019 |
| Serum creatinine, mg/dL | 8.8±2.3 | 8.8±2.3 | 0.985 | 0.012 |
| Serum albumin, g/dL | 3.6±0.5 | 3.6±0.4 | 0.562 | 0.030 |
| Serum total cholesterol, mg/dL | 152 (127–184) | 150 (125–173) | 0.818 | 0.011 |
| Serum C-reactive protein, mg/dL | 0.15 (0.06–0.43) | 0.17 (0.09–0.54) | 0.596 | 0.042 |
| Corrected serum Ca, mg/dL | 9.3±0.9 | 9.3±0.8 | 0.924 | 0.008 |
| Serum phosphate, mg/dL | 4.7±1.2 | 4.6±1.8 | 0.088 | 0.101 |
| Serum alkaline phosphatase, U/L | 298±156 | 293±195 | 0.682 | 0.031 |
| Serum PTH (intact assay), pg/mL | 106 (53-201) | 86 (41-195) | 0.935 | 0.003 |
| **Medications, %** |  |  |  |  |
| Use of ESAs | 88 | 86 | 0.282 | 0.064 |
| Use of anti-hypertensives | 61 | 63 | 0.946 | 0.004 |
| Use of VDRAs | 64 | 64 | 0.946 | 0.018 |
| Use of phosphate-binders |  |  |  |  |
| Sevelamer hydrochloride | 0 | 27 | <0.001 | 0.858 |
| Ca-containing binders | 0 | 86 | <0.001 | 3.352 |

Data are expressed as the mean ± standard deviation, median (interquartile range), or percentage, depending on the nature of the variable. Unpaired *t*-test, chi-square test, or Wilcoxon signed-rank test was used to compare the two groups. Standardized difference was also calculated. Propensity score was created by multivariable logistic regression analyses using covariates listed in the methods section. Comorbidity included history of cardiovascular events, bone fracture, and parathyroidectomy. A two-tailed *P*-value less than 0.05 was considered statistically significant. Abbreviations: Ca, calcium; ESAs, erythropoiesis-stimulating agents; nPCR, normalized protein catabolic rate; PTH, parathyroid hormone; VDRAs, vitamin D receptor activators.

# Supplementary Table 2. Associations between dose of P-binders and the risk of infection-related and all-cause mortality

|  | Group 1  (P-binder; 0 mg/day) | Group 2  (0 mg/day< P-binder< 2000 mg/day)  (n=1084) | Group 3  (2000 mg/day≤ P-binder≤ 15000 mg/day)  (n=1325) | *P* for trend |
| --- | --- | --- | --- | --- |
| Infection-related mortality | 1 (reference) | 0.67 (0.42-1.07) | 0.53 (0.29-0.96) | 0.035 |
| All-cause mortality | 1 (reference) | 0.78 (0.52-0.90) | 0.68 (0.52-0.90) | 0.006 |

Patients were divided into three groups based on the dose of P-binders: Group 1, patients without P-binders use; Group 2, patients whose dose of P-binders were between 0 and 2000 mg/day; Group 3, patients whose dose of P-binders were between 2000-15000 mg/day. The dose of P-binders were expressed as the dose of calcium-based P-binders. In the current study, 500 mg of calcium- based P-binders was equivalent to 750 mg/day of sevelamer hydrochloride. The hazards ratios and 95% confidence interval of the incidence of infection-related and all-cause mortality were estimated by multivariable-adjusted Cox proportional hazards model. In the multivariable model, age, sex, presence of diabetes mellitus and comorbidity, dialysis history, dialysis time per session, Kt/V for urea, normalized protein catabolic rate, body mass index, cardiothoracic ratio, systolic blood pressure, blood hemoglobin level, serum levels of urea nitrogen, creatinine, albumin, total cholesterol, C-reactive protein, corrected calcium, phosphate, alkaline phosphatase, and parathyroid hormone, and use of erythropoiesis-stimulating agents, anti-hypertensives, and vitamin D receptor activators were included. Data are expressed as hazards ratio (95% confidence interval). A two-tailed *P*-value less than 0.05 was considered statistically significant.

**Supplemental Table S3**. Associations between treatment with phosphate-binders and the risk of infection-related and all-cause mortality in patients without hypophosphatemia

|  | Infection-related mortality | | All-cause mortality | |
| --- | --- | --- | --- | --- |
| PS-adjusted model | HR (95% CI) | *P*-value | HR (95% CI) | *P*-value |
| PS-matched model (1:1, n=904) | 0.58 (0.34-0.99) | 0.047 | 0.73 (0.56-0.94) | 0.017 |
| PS-stratification (n=2800) | 0.58 (0.34-0.99) | 0.044 | 0.73 (0.57-0.95) | 0.017 |
| PS-adjusted regression model (n=2800) | 0.64 (0.40-1.01) | 0.057 | 0.74 (0.59-0.92) | 0.007 |
| IPTW model (n=2800) | 0.66 (0.42-1.04) | 0.073 | 0.68 (0.53-0.91) | <0.001 |

Patients with baseline serum phosphate level ≤ 3 mg/dL were excluded in the analysis. The HR was estimated using Cox proportional hazard model. In the multivariable model, age, sex, presence of diabetes mellitus and comorbidity, dialysis history, dialysis time per session, Kt/V for urea, normalized protein catabolic rate, body mass index, cardiothoracic ratio, systolic blood pressure, blood hemoglobin level, serum levels of urea nitrogen, creatinine, albumin, total cholesterol, C-reactive protein, corrected calcium, phosphate, alkaline phosphatase, and parathyroid hormone, and use of erythropoiesis-stimulating agents, anti-hypertensives, and vitamin D receptor activators were included. PS was created by logistic regression analysis using all the parameters listed here. A two-tailed *P*-value less than 0.05 was considered statistically significant. Abbreviations: CI, confidence interval; HR, hazards ratio; IPTW, inverse probability of treatment weighting; PS, propensity score.
